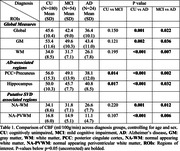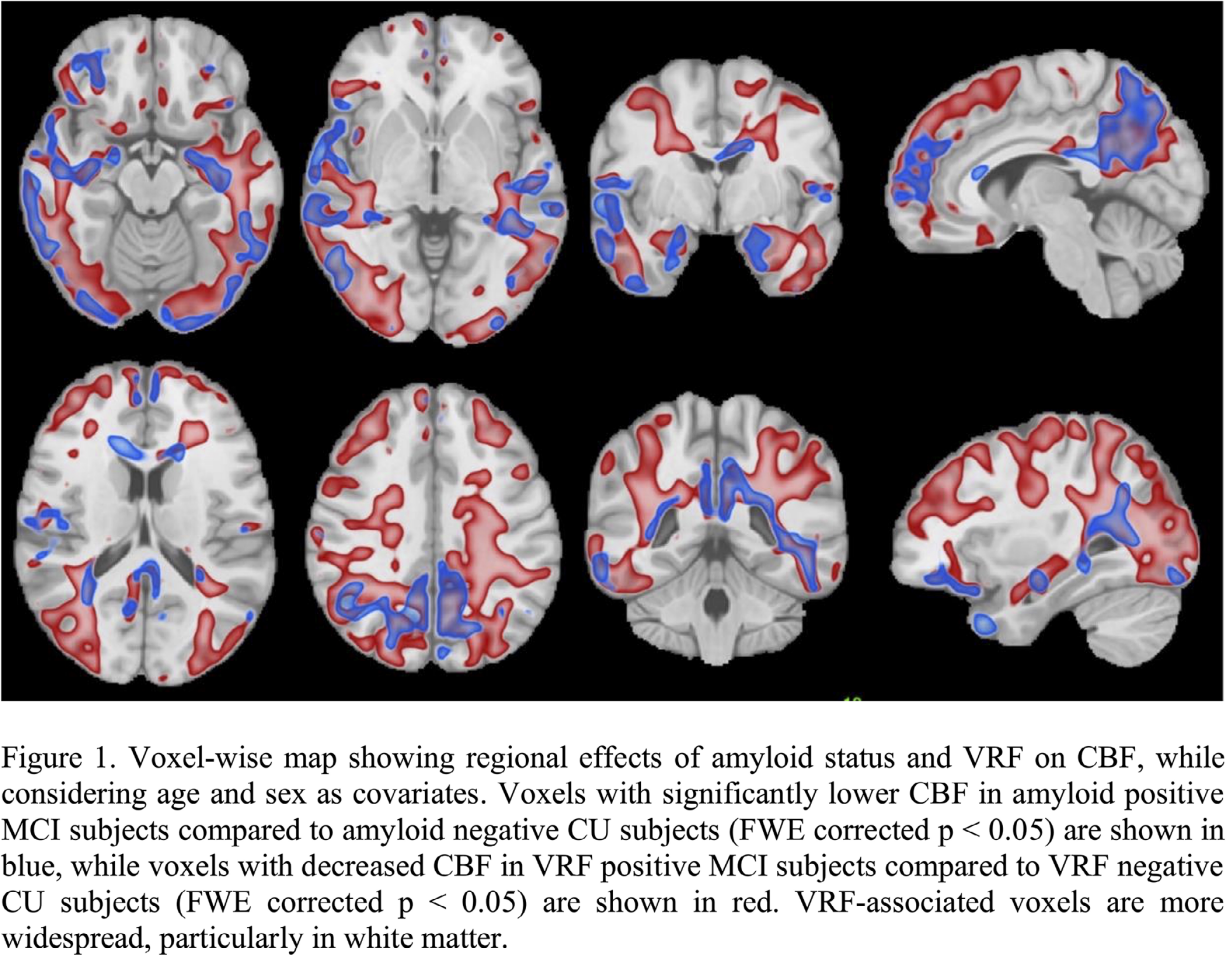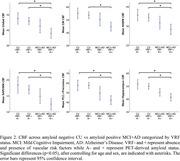# Effects of Amyloid Pathology and Vascular Risk Factors on Regional Cerebral Blood Flow Patterns

**DOI:** 10.1002/alz.093000

**Published:** 2025-01-09

**Authors:** Mohammad Taghvaei, Shokufeh Sadaghiani, Banafsheh Shakibajahromi, Pulkit Khandelwal, Sandhitsu R. Das, Ilya M. Nasrallah, Paul A. Yushkevich, David A Wolk, John A. Detre, Sudipto Dolui

**Affiliations:** ^1^ Department of Neurology, University of Pennsylvania, Philadelphia, PA USA; ^2^ Penn Image Computing and Science Laboratory (PICSL), University of Pennsylvania, Philadelphia, PA USA; ^3^ Department of Radiology, University of Pennsylvania, Philadelphia, PA USA; ^4^ Penn Alzheimer’s Disease Research Center, University of Pennsylvania, Philadelphia, PA USA

## Abstract

**Background:**

Regional cerebral blood flow (CBF) can be noninvasively quantified using arterial spin labeled (ASL) perfusion MRI. In Alzheimer’s disease (AD) and mild cognitive impairment (MCI), hypoperfusion typically occurs in precuneus, posterior cingulate cortex (PCC), and hippocampus. Small vessel disease (SVD), a systemic disorder that commonly underlies vascular cognitive impairment, also causes brain hypoperfusion. Many dementia patients demonstrate both neurodegenerative and vascular pathologies, which may interact to accelerate cognitive decline. Here we used advanced background‐suppressed 3D ASL to assess CBF variations across the AD spectrum and in relation to risk factors for AD and SVD.

**Method:**

Data from 258 participants comprising 180 cognitively unimpaired (CU), 54 MCI, and 24 AD participants, diagnosed by clinical consensus, were obtained from the Penn Alzheimer Disease Research Center. ASL CBF was quantified in gray matter (GM), white matter (WM), AD‐associated cortical regions of PCC+precuneus and hippocampus. We also considered normal‐appearing WM (NA‐WM) and periventricular WM (NA‐PVWM) in which voxels containing T2 white matter hyperintensities were excluded. PVWM is supplied exclusively by microvessels and may be a sensitive region for CBF reductions in SVD despite its small size. Global and regional CBF was compared across diagnostic groups. Furthermore, the influence of PET‐derived amyloid status and vascular risk factors (VRF: presence of any of hypertension, diabetes, obesity, hypercholesteremia, and smoking) on CBF were also evaluated.

**Result:**

Across the AD spectrum, CBF was lower in AD‐associated cortical regions. AD also exhibited reduced CBF in GM, WM, NA‐WM, and NA‐PVWM (Table 1). Voxel‐wise comparisons of CBF contrasting amyloid‐positive or VRF‐positive MCI with corresponding amyloid‐negative or VRF‐negative CU showed that VRF effects were more widely distributed in GM and WM than amyloid status effects (Figure 1). There was a trend of reduced regional CBF with VRF‐positivity in amyloid‐positive AD+MCI suggesting an additive effect of VRF with amyloid and diagnosis (Figure 2).

**Conclusion:**

Putative SVD regions showed diagnosis‐related CBF changes comparable in magnitude to typical AD cortical regions that may reflect vascular effects. Vascular risk was associated with more spatially distributed CBF reductions than amyloid status and may reflect its additive effect with amyloid on cognitive decline.